# Andrographolide Suppresses Influenza A Virus-Induced Pyroptosis via PI3K/AKT-Mediated Caspase-3/GSDME Inactivation

**DOI:** 10.3390/biomedicines14040887

**Published:** 2026-04-13

**Authors:** Wen Yang, Qi He, Zhen Sun, Xiaochang Zhang, Qingyu Li, Changdong Zhou, Yuke Cui, Zhenqiao Wei, Jingqi Shi, Chenhui Wang, Yuanyuan Jiao, Liang Guo, Yaling Xing, Shengqi Wang

**Affiliations:** 1 Bioinformatics Center of AMMS, Beijing 100850, China; 15589965413@163.com (W.Y.);; 2 Pingyuan Laboratory, Xinxiang 453007, China; 3 State Key Laboratory of Kidney Diseases, Beijing 100853, China

**Keywords:** andrographolide, *influenza A virus*, PI3K/AKT signaling pathway, pyroptosis

## Abstract

**Background/Objectives**: *Influenza A virus* (IAV) infection triggers robust inflammation and acute lung injury. Andrographolide, a primary active compound from *Andrographis paniculata*, can mitigate IAV-induced inflammation; however, its precise mechanisms remain poorly elucidated. This study aimed to define its host-directed protective effects and molecular mechanisms. **Methods**: We used a lethal IAV (H1N1, PR8) model in *BALB/c* mice and infected A549 cells. Survival, lung pathology, cytokines, and viral titers were measured. Lung RNA sequencing identified dysregulated signaling pathways. PI3K/AKT and pyroptosis pro-teins were analyzed by Western blot. The PI3K/AKT axis was functionally validated with the AKT inhibitor in vivo and AKT1 siRNA in vitro. **Results**: Andrographolide improved survival, attenuated body weight loss, and reduced lung pathology and inflammatory cytokine levels in IAV-infected mice, without exhibiting direct antiviral activity. Consistent with the in vivo findings, andrographolide enhanced cell viability and suppressed cytokine secretion in infected cells. RNA sequencing revealed marked upregulation of the PI3K/AKT signaling pathway in the lungs of treated mice, as confirmed by increased PI3K and AKT phosphorylation. Furthermore, andrographolide downregulated the expression of key pyroptosis-executing proteins, including cleaved caspase-3 and the gasdermin E (GSDME) N-terminal fragment. These protective effects were substantially abrogated by an AKT inhibitor and AKT1 siRNA. **Conclusions**: These findings reveal a novel host-directed mechanism by which andrographolide alleviates IAV-induced immunopathology by activating the PI3K/AKT pathway, thereby suppressing caspase-3/GSDME-dependent pyroptosis. Thus, this axis represents a promising target for controlling excessive inflammation in severe influenza.

## 1. Introduction

The *Influenza A virus* (IAV) poses a continuous threat to global public health. Endemic or seasonal IAV infections result in an estimated 3 to 5 million cases of severe illness and up to 650,000 deaths annually worldwide [1]. Historically, IAV pandemics, such as those caused by the 1918 and 2009 H1N1 strains, have had a profound global impact [2,3,4]. Viral pneumonia and acute respiratory distress syndrome (ARDS) are the most frequent lethal outcomes of severe IAV infection [5,6]. It is well-established that excessive pulmonary inflammatory responses significantly drive IAV-associated morbidity and mortality [7,8].

Although antiviral drugs targeting the virus, including neuraminidase inhibitors, M2 ion channel blockers, and polymerase inhibitors, have been developed and approved for clinical use [9,10,11,12], the emergence of drug-resistant viral strains remains a major clinical challenge [13]. This limitation has spurred growing interest in host-directed antiviral therapies. Host signaling pathways and cellular factors hijacked by viruses represent a broad spectrum of potential targets for combating IAV infection [14,15,16,17,18,19]. There is an urgent need to develop effective antiviral agents with broad-spectrum activity and a reduced risk of resistance.

Natural compounds derived from medicinal plants play a crucial role in antiviral agent discovery [20,21,22,23,24,25,26,27]. Andrographolide, a diterpenoid lactone from *Andrographis paniculata*, exhibits broad-spectrum antiviral activity against viruses, including *severe acute respiratory syndrome coronavirus 2* (SARS-CoV-2) [28,29,30]. Furthermore, andrographolide exhibits demonstrated antibacterial, anti-inflammatory, and antitumor properties [31,32,33,34,35,36]. Previous studies have demonstrated that andrographolide exerts anti-IAV effects through mechanisms that modulate Retinoic acid-inducible gene I(RIG-I)-related signaling pathways [37]. Other research groups have shown that andrographolide suppresses inflammation by downregulating NACHT, LRR and PYD domains-containing protein 3 (NLRP3) inflammasome expression while upregulating that of the Signal Transducer and Activator of Transcription 3 (STAT3) and Nuclear factor erythroid 2-related factor 2 (Nrf2) pathways [38,39,40,41]. However, whether andrographolide exerts additional, particularly host-targeted mechanisms that counteract inflammation warrants further investigation.

Accumulating evidence indicates that the Phosphatidylinositol 3-kinase/AKT serine/threonine kinase (PI3K/AKT) signaling pathway is closely associated with IAV-triggered inflammation [42]. AKT activation can modulate various cellular processes that are critical for inflammation control, including the inhibition of gasdermin E (GSDME)-mediated pyroptosis [43,44]. Pyroptosis, a highly inflammatory form of programmed cell death, has recently been recognized as a critical driver of IAV pathogenesis [45,46]. Excessive pyroptosis in lung epithelial cells leads to the release of proinflammatory cytokines and cellular contents, exacerbating lung injury and potentially contributing to the occurrence of cytokine storms [47,48,49,50]. Given that the PI3K/AKT axis is a known regulator of both inflammatory responses and cell death, and considering the established anti-inflammatory properties of andrographolide, we hypothesized that andrographolide protects against IAV-induced lung injury by activating the PI3K/AKT pathway, thereby suppressing pyroptosis.

To systematically decipher the mechanism underlying the protective effect of andrographolide against IAV, we designed a sequential investigational strategy: (a) confirming its protective efficacy in vivo and in vitro; (b) employing unbiased RNA sequencing to screen for key dysregulated host pathways; (c) validating the activation of the prioritized PI3K/AKT pathway and its functional consequence on suppressing GSDME-mediated pyroptosis; and (d) definitively establishing the pathway’s necessity using combined pharmacological and genetic inhibition approaches.

In this study, we demonstrated that andrographolide protects against inflammatory injury in IAV-infected mice and cells without inhibiting viral replication. We found that andrographolide attenuates inflammation and caspase-3/GSDME-dependent pyroptosis by activating the PI3K/AKT signaling pathway. These findings elucidate a novel mechanism of action for andrographolide and provide a scientific foundation for its potential application in mitigating excessive inflammatory responses in influenza and, potentially, other diseases.

## 2. Materials and Methods

### 2.1. Virus

The *influenza A virus A/Puerto Rico/8/34* (H1N1; PR8) was amplified in embryonated eggs obtained from specific pathogen-free (SPF) chickens (9-day-old) through incubation at 37 °C for 48 h. The allantoic fluid was then harvested, clarified by centrifugation at 1000× *g* for 10 min, aliquoted, and stored at −80 °C. The median lethal dose (LD_50_) in mice and the median tissue culture infective dose (TCID_50_) in MDCK cells were determined using the Reed–Muench method.

### 2.2. Cells

Cell culture was performed using Madin–Darby canine kidney (MDCK) cells and human alveolar epithelial A549 cells. The A549 and MDCK cells were obtained from the American Type Culture Collection (ATCC, Manassas, VA, USA), with catalog numbers CCL-185™ and CCL-34™, respectively. Both cell types were grown in Dulbecco’s Modified Eagle Medium/F-12, supplemented with 10% fetal bovine serum (FBS) and a 1% penicillin–streptomycin solution. A standard humidified incubator providing 37 °C and 5% CO_2_ was used for maintaining all cultures.

### 2.3. Animals

SPF male *BALB/c* mice (weighing approximately 20 g) were purchased from Charles River Laboratories (License No. SCXK-(Jing) 2020-0006, Wilmington, MA, USA) and housed in the Animal Facility of the AMMS under standard conditions. Mice were randomly assigned to the four experimental groups (Efficacy evaluation: Control *n* = 15, H1N1-infected *n* = 13, H1N1 + Andrographolide *n* = 21, H1N1 + Oseltamivir *n* = 17. AKT Inhibition assay: Control *n* = 9, H1N1-infected *n* = 10, H1N1 + Andrographolide *n* = 10, H1N1+Andrographolide + MK2206 *n* = 10) using a computer-generated random number sequence to ensure each animal had an equal chance of being assigned to any group. Blinding was implemented during outcome assessment and data analysis to minimize bias. The investigators responsible for measuring outcomes and performing statistical analysis were unaware of the group allocations.

To establish the infection model, mice were anesthetized with sodium pentobarbital (50 mg/kg) and inoculated intranasally (i.n.) with 4 LD_50_ of the PR8 virus in sterile phosphate-buffered saline (PBS). Mice in the control group received a daily oral gavage of PBS. Treatment groups received daily intragastric administrations of either andrographolide (150 mg/kg; Meilun Bio, Dalian, China, #MB2185-1) or oseltamivir phosphate (20 mg/kg; Meilun Bio, #MB1537-1) from day 0 to day 4 post-infection. We strictly followed predefined humane endpoints to minimize animal suffering. Euthanasia was performed if mice exhibited: ≥25% body weight loss, severe lethargy/inability to feed, severe respiratory distress, or irreversible moribundity. Mice were sacrificed on days 2, 4, and 15 post-infection. Bronchoalveolar lavage fluid (BALF) cytokine levels were measured by an enzyme-linked immunosorbent assay (ELISA). Lung tissues were processed for Western blotting, quantitative reverse transcription polymerase chain reaction (qRT-PCR), and histopathological examination. For the AKT inhibition study, mice were pretreated with the AKT inhibitor MK-2206 (Selleck, Houston, TX, USA, #S1078) for 4 days prior to infection, with treatment continuing for 4 days post-infection.

### 2.4. RNA-Seq Analysis

The experimental timeline proceeded as follows: mice were euthanized on the fourth day following infection, and their lung tissues were immediately collected. Following this, total RNA was extracted from the homogenized lung samples utilizing TRIzol reagent (Invitrogen, Carlsbad, CA, USA, 15596018CN). Subsequently, RNA-seq libraries were prepared specifically from high-integrity RNA that passed quality assessments, which is a critical step for reliable transcriptomic profiling (RNA Integrity Number > 7.0) by Annoroad Gene Technology Co., Ltd. (Beijing, China). Briefly, after second-strand cDNA synthesis, libraries were constructed using a heat-labile uracil-DNA glycosylase enzyme protocol (New England Biolabs, Ipswich, MA, USA) and amplified by PCR; fragments of 300 ± 50 bp were selected.

Sequencing reads were aligned to the *Mus musculus* reference genome, and gene expression levels were quantified as fragments per kilobase of transcript per million mapped reads. The mapped reads of each sample were assembled using StringTie (https://ccb.jhu.edu/software/stringtie) (accessed on 21 April 2025) with default parameters. Then, all transcriptomes from all samples were merged to reconstruct a comprehensive transcriptome using gffcompare (https://github.com/gpertea/gffcompare/) (accessed on 6 May 2025). After the final transcriptome was generated, StringTie was used to estimate the expression levels of all transcripts. StringTie was used to calculate expression levels for mRNAs by calculating FPKM. The differentially expressed mRNAs were selected with fold change > 2 or fold change < 0.5 and with parametric F-test comparing nested linear models (*p*-value < 0.05) by the R package edgeR (https://bioconductor.org/packages/release/bioc/html/edgeR.html) (accessed on 24 May 2025).

### 2.5. qRT–PCR

Total RNA from lungs or cells was extracted using a commercial kit (TIANGEN, Beijing, China, #DP451). RNA purity and concentration were determined by spectrophotometry (A260/A280). cDNA was synthesized from 800 ng of total RNA using a reverse transcription kit (TIANGEN, Beijing, China, #FP209-02). qRT-PCR was performed using SuperReal PreMix Plus SYBR Green (TIANGEN, Beijing, China, #KR11-02) on an ABI 7500 instrument (Applied Biosystems, Carlsbad, CA, USA). The amplification protocol comprised an initial 3-min denaturation step at 95 °C to activate the DNA polymerase, followed by 40 cycles of PCR. Each cycle included a 20-s denaturation at 95 °C and a 32-s combined annealing/extension phase at 60 °C. To quantify gene expression, the comparative Ct (2^(−ΔΔCt)^) method was employed, with GAPDH serving as the internal reference gene for data normalization. The primer sequences are provided in Supplemental Data (Table S1).

### 2.6. Western Blotting

Protein extraction from lung tissues and cultured cells was conducted on ice using RIPA lysis buffer supplemented with protease and phosphatase inhibitors. Following centrifugation at 4 °C, the supernatant was collected, and total protein concentration was measured with a BCA assay kit (Thermo Scientific, Waltham, MA, USA, #23227). Equal protein quantities (60 µg per lane) were resolved by SDS-PAGE and electrophoretically transferred to PVDF membranes (Millipore, Burlington, MA, USA, IPVH15150). The membranes were blocked with 5% non-fat dry milk for 1 h at room temperature, then probed with primary antibodies at 4 °C overnight. After washing, membranes were incubated with HRP-conjugated secondary antibodies for 1 h at room temperature. Signal detection was performed using an ECL substrate (Thermo Scientific, Waltham, MA, USA, #32106), and images were acquired with a Fusion FX system (Vilber Lourmat, Collégien, France).

Primary antibodies against p-AKT (Ser473 4060S) and AKT (4691) were purchased from Cell Signaling Technology (Beverly, MA, USA). NP(SB11675-T62) and HA (SB11684-R107) were purchased from Sino Biological (Beijing, China). p-PI3K (p85p65 AF3242), PI3K (p85p65 AF6242), p-MAPK (AF4001), MAPK (AF6456), and GSDME (DF9705) were purchased from Affinity Biosciences (Cincinnati, OH, USA). Caspase-3 (184787), GSDMD (abcam, ab209845, GSDME (abcam, ab215191), caspase-1 (abcam, ab207802), Goat Anti-Rabbit IgG H&L (HRP) (abcam, ab205718), Goat Anti-Mouse IgG H&L (HRP) (St. Louis, MO, USA) (abcam, 205719), and β-actin (ab8227) were purchased from abcam (Cambridge, UK).

### 2.7. ELISA

Levels of IL-1β, IL-6, and TNF-α in BALF were measured using commercial ELISA kits (DAKEWE, Shenzhen, China, #1210122, #1210602, #1217202), per the manufacturer’s instructions. Quantification was performed by measuring the optical density (OD) at 450 nm using a microplate reader (Epoch, BioTek, Winooski, VT, USA).

### 2.8. Immunohistochemistry (IHC)

Lung tissue samples were fixed with 4% paraformaldehyde, embedded in paraffin, and sliced into 5 µm sections. Following deparaffinization and rehydration, antigen retrieval was carried out using a citrate buffer (pH 6.0). To eliminate endogenous peroxidase activity that could cause background interference, the sections were subsequently treated with a 3% hydrogen peroxide solution. Nonspecific binding was blocked with goat serum. The sections were then incubated overnight at 4 °C with an anti-cleaved-caspase-3 antibody (abcam, ab32042, Cambridge, UK 1:200), followed by a secondary antibody. DAB development was employed for visualization.

### 2.9. Cell Viability Assay

Cell viability was assessed using a CCK-8 kit (Beyotime, Shanghai, China, #C0038). MDCK or A549 cells were seeded in 96-well plates (2 × 10^4^ cells/well). After 24 h, the cells were infected with IAV at 100 TCID_50_ and treated with andrographolide. After 48 h, 10 µL of CCK-8 reagent was added to each well, and the plates were incubated for 1 h. Absorbance was measured at 450 nm.

### 2.10. Plaque Reduction Assay

For the plaque assay, MDCK cells forming a monolayer in 12-well plates were first subjected to infection with lung homogenate supernatants from different experimental groups of mice for 2 h at 37 °C. The viral inoculum was then replaced by an agar-overlay medium (0.6% agar, 2% FBS). A 48-h incubation followed. Subsequently, the cells were fixed with 10% formalin and stained using a 1% crystal violet solution. The plaques that formed were finally counted and photographs were taken for record keeping.

### 2.11. Multiplexed Immunofluorescence

For immunostaining, the sections were incubated in 3% H_2_O_2_ for 20 min followed by deparaffinization and heat-mediated antigen retrieval treatment and incubated in blocking buffer (3% BSA in PBS supplemented with 0.1% Triton X-100) at room temperature for 1 h. Sections were incubated with primary antibody for 2 h, followed by detection using HRP-conjugated secondary antibody and TSA-fluorophores. The primary and secondary antibodies were eliminated by heating the slides. CD68 (CST, # 97778, 1:400) and Ly-6G (CST, # 87048, 1:300) were sequentially detected. Vectashield containing DAPI nuclear counterstain was used to mount the sections. The slices were imaged using the Olympus FVS300 slide scanner (Tokyo, Japan).

### 2.12. Lactate Dehydrogenase (LDH) Release Assay

To determine cytotoxicity, the release of LDH was measured with a commercial kit (Beyotime, Shanghai, China, # C0016). After cells in 96-well plates received the indicated treatments, the assay was conducted according to the manufacturer’s instructions. The included lysis solution was utilized to induce maximum LDH release for calibration.

### 2.13. Propidium Iodide (PI) Staining

A549 cells were infected with H1N1. At specified time points post-infection, cells were stained with annexin V and PI using an Annexin V-FITC Apoptosis Detection Kit (Beyotime, Shanghai, China, # C1062L), in accordance with the manufacturer’s protocol.

### 2.14. siRNA Assay

To knock down target gene expression, AKT1-specific siRNAs, along with a non-targeting scramble siRNA (used as a negative control), were commercially synthesized (Sangon Biotech, Shanghai, China). Transfection of A549 cells was carried out with Lipofectamine 3000 reagent, strictly following the provided protocol. The successful silencing of target proteins was confirmed via Western blot analysis. The corresponding siRNA sequences are listed in Supplementary Materials Table S2.

### 2.15. Statistical Analysis

The data are presented as mean ± standard error of the mean. Differences among multiple groups were analyzed by one-way or two-way ANOVA, as appropriate. Survival curves were compared using the log-rank test. Analyses were performed using GraphPad Prism 8 (version 8.0.2), with *p* < 0.05 considered to indicate significance.

## 3. Results

### 3.1. Andrographolide Conferred Protection Against IAV Infection In Vivo and In Vitro

**Figure 1:**
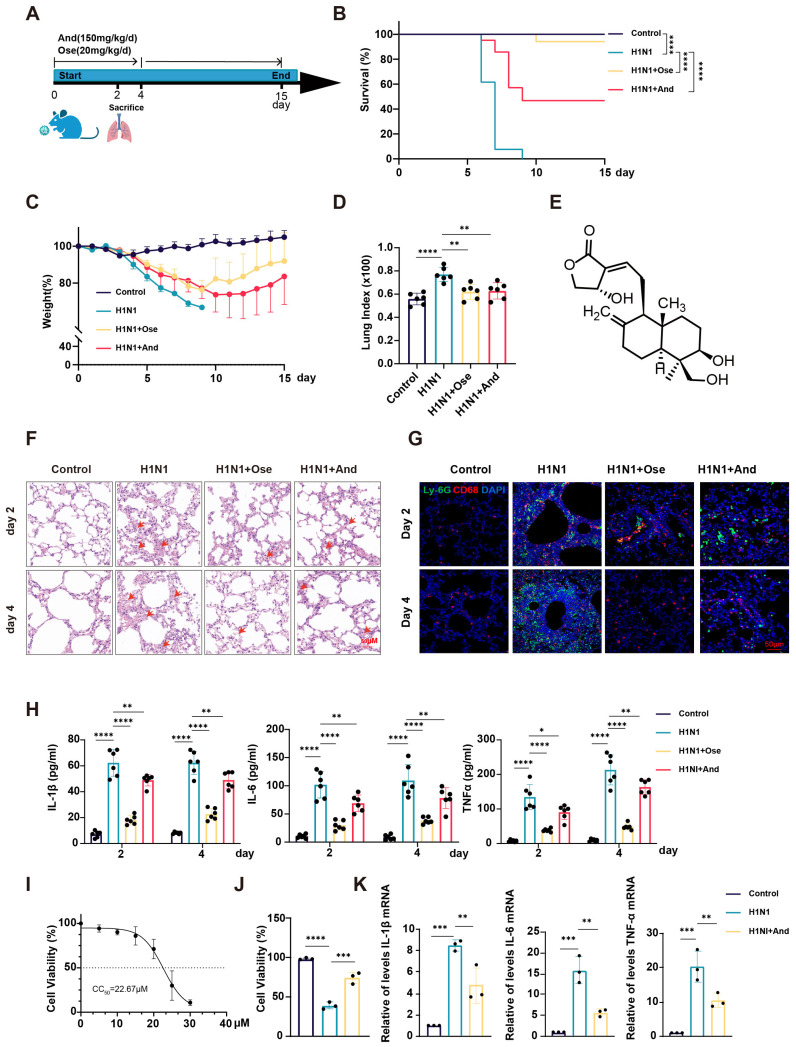
Andrographolide conferred protection against *influenza A Virus* infection in vivo and in vitro. (**A**) Schematic of the experimental timeline. BALB/c mice were infected intranasally (i.n.) with 4 LD_50_ of IAV on day 0. Mice were treated daily by intragastric administration (i.g.) with andrographolide (150 mg/kg) or Ose (20 mg/kg) from day 0 to day 4. (**B**,**C**) Mouse survival rates (%) (**B**) and body weight changes (%) (**C**) were monitored for 15 days post-infection (dpi). (Control *n* = 15, H1N1-infected *n* = 13, H1N1 + Andrographolide *n* = 21, H1N1 + Oseltamivir *n* = 17). (**D**) Lung index (lung weight/body weight) as measured on day 4. (*n* = 6). (**E**) Chemical structure of andrographolide. (**F**) Representative H&E-stained lung tissue sections. Arrows indicate alveolar hemorrhage and thickened alveolar walls. Scale bar, 50 µm. (*n* = 3). (**G**) Representative images of neutrophils (Ly6G) and macrophages (CD68) in lung tissues from different groups. Scale bar, 50 µm. (*n* = 3). (**H**) Levels of IL-6, TNF-α, and IL-1β in BALF measured by ELISA on days 2 and 4 post-infection. (*n* = 6). (**I**) Cytotoxicity of And in A549 cells determined by CCK-8 assay. (*n* = 3). (**J**) Cell viability of A549 cells infected with IAV and treated with andrographolide (15 μM), assessed by CCK-8 assay. (*n* = 3). (**K**) mRNA expression levels of IL-6, TNF-α, and IL-1β in A549 cells treated with andrographolide (15 μM) post-IAV infection, measured by qRT–PCR. (*n* = 3). The results are shown as the mean ± standard error of the mean. * *p* < 0.05, ** *p* < 0.01, *** *p* < 0.001, **** *p* < 0.0001. And represents andrographolide; Ose represents oseltamivir phosphate.

To evaluate the therapeutic potential of andrographolide against influenza, IAV-infected mice were treated with andrographolide (150 mg/kg/day) or oseltamivir (positive control) and monitored for 15 days (Figure 1A). Kaplan–Meier survival analysis showed that all mice in the H1N1-infected group succumbed within 9 days post-infection. In contrast, andrographolide treatment conferred a 47.6% survival rate, and oseltamivir achieved a 94.1% survival rate (Figure 1B). Andrographolide also significantly attenuated IAV-induced body weight loss compared with the H1N1 group, with the lowest weight occurring on day 10 post-infection, 1 day later than in the oseltamivir group (day 9) (Figure 1C). These data demonstrate that andrographolide improves survival and ameliorates weight loss in IAV-infected mice.

Consistent with the body weight changes, the H1N1 group exhibited more severe clinical symptoms. Andrographolide treatment significantly reduced the lung index on day 4 post-infection compared with that in the H1N1 group (Figure 1D). The histopathological examination (H&E) of lung tissues revealed that IAV infection caused alveolar collapse, interstitial thickening, and inflammatory cell infiltration, all of which were markedly alleviated by andrographolide treatment (Figure 1F). IAV infection induced substantial infiltration of both CD68 macrophages and Ly6G^+^ neutrophils into lung tissue, which was significantly reduced by andrographolide treatment (Figure 1G). Furthermore, the ELISA analysis of BALF collected on days 2 and 4 post-infection showed that andrographolide significantly suppressed the IAV-induced elevation of IL-1β, IL-6, and TNF-α levels (Figure 1H). Collectively, these results indicate that andrographolide attenuates the inflammatory response in the lungs of IAV-infected mice.

We next assessed the effects of andrographolide in vitro. Per the CCK-8 assay, the half-maximal cytotoxic concentration (CC_50_) of andrographolide in A549 cells was 22.67 µM (Figure 1I). Using a non-toxic concentration (15 µM), we found that andrographolide significantly enhanced cell viability following IAV infection (Figure 1J). qRT-PCR confirmed that andrographolide treatment significantly downregulated the mRNA expression of key inflammatory cytokines (IL-1β, IL-6, and TNF-α) in IAV-infected A549 cells (Figure 1K). Furthermore, andrographolide inhibited both virus-induced LDH release and cell death, the latter assessed by PI staining (Figure S1A,B). These in vitro findings corroborated the anti-inflammatory effects of andrographolide observed in vivo.

### 3.2. Andrographolide Did Not Affect IAV Replication

**Figure 2:**
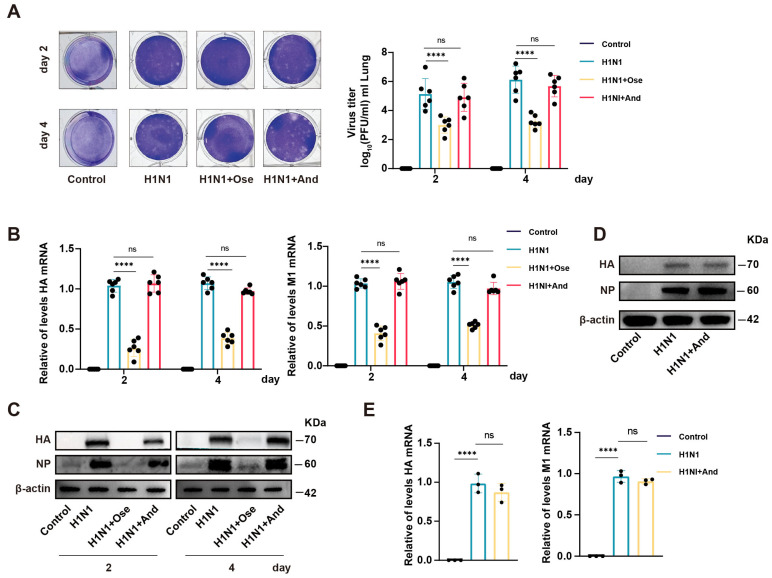
Andrographolide did not affect *influenza A Virus* replication. (**A**) Plaque formation assay in MDCK cells using lung homogenates from mice on days 2 and 4 post-infection. (*n* = 6). (**B**) qRT-PCR analysis of viral M1 and HA gene expression in mouse lungs on days 2 and 4 post-infection. (*n* = 6). (**C**) Western blotting of viral HA and NP protein levels in mouse lungs on days 2 and 4 post-infection. (*n* = 3). (**D**) Western blot analysis of viral HA and NP protein levels in IAV-infected A549 cells treated with andrographolide (15 μM). (*n* = 3). (**E**) qRT-PCR analysis of viral M1 and HA gene expression in IAV-infected A549 cells treated with andrographolide (15 μM). (*n* = 3). The results are expressed as the mean ± standard error of the mean. **** *p* < 0.0001. ns indicates no significance.

Given the protective effects of andrographolide, we investigated whether it directly inhibited viral replication. Plaque reduction assays showed no significant difference in viral plaque numbers between the H1N1 and andrographolide-treated groups on days 2 and 4 post-infection, indicating a lack of direct antiviral activity (Figure 2A). Consistent with this, qRT–PCR revealed no significant changes in the relative expression levels of viral M1 and HA genes in the lungs of andrographolide-treated mice compared with those in the H1N1 group (Figure 2B). Western blotting further confirmed that andrographolide treatment did not alter the protein levels of HA and NP (Figure 2C). Similarly, in vitro experiments showed that andrographolide had no significant effect on viral protein (HA, NP) or gene (M1, HA) expression in infected A549 cells (Figure 2D,E). These results demonstrate that the protective effect of andrographolide is not mediated by IAV replication inhibition; instead, it likely involves the modulation of host inflammatory responses.

### 3.3. Andrographolide Activated the PI3K/AKT Signaling Pathway and Inhibited IAV-Induced Pyroptosis

**Figure 3:**
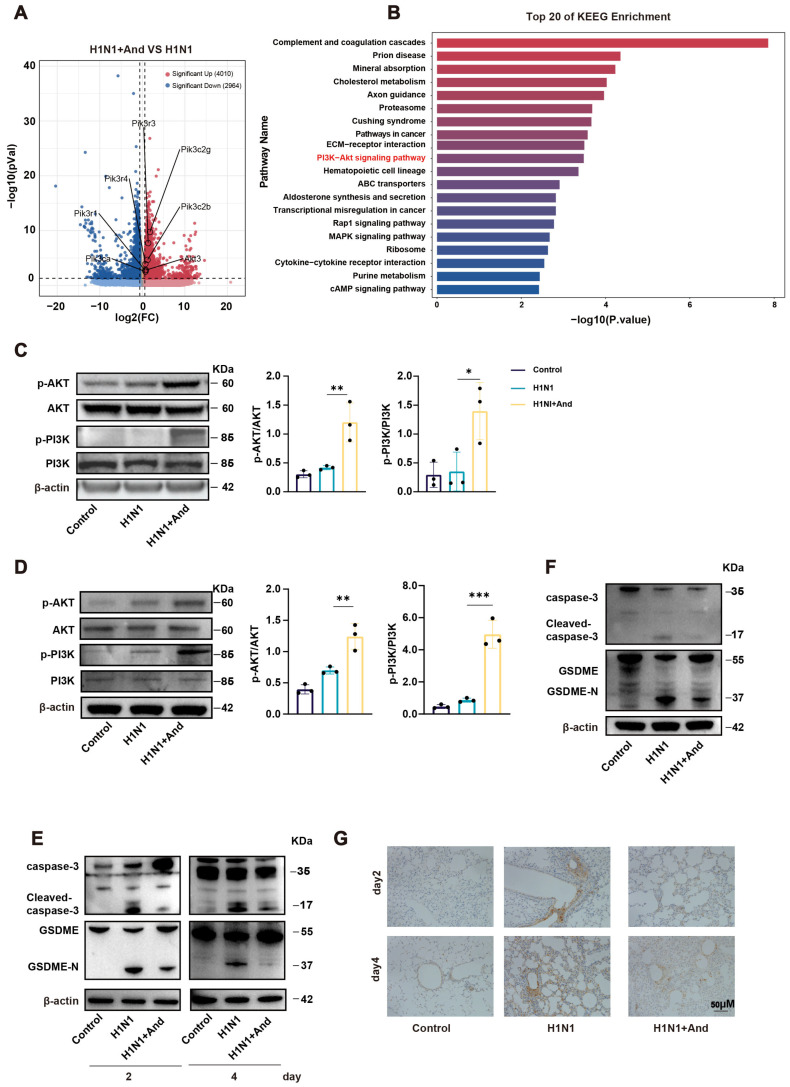
Andrographolide activated the PI3K/AKT signaling pathway and inhibited *influenza A Virus* -induced pyroptosis. (**A**) Volcano plot displaying differentially expressed genes in lung tissues between H1N1 + And and H1N1 groups on day 4 post-infection. (*n* = 4). (**B**) Top 20 enriched KEGG pathways from the RNA-seq analysis. (*n* = 4). (**C**) Western blotting of key proteins in the PI3K/AKT signaling pathway in mouse lung tissues. (*n* = 3). (**D**) Western blot analysis of key proteins in the PI3K/AKT signaling pathway in IAV-infected A549 cells. (*n* = 3). (**E**) Western blotting of GSDME, GSDME-N, caspase-3, and cleaved caspase-3 levels in mouse lung tissues. (*n* = 3). (**F**) Western blotting of GSDME, GSDME-N, caspase-3, and cleaved caspase-3 levels in IAV-infected A549 cells. (*n* = 3). (**G**) Representative IHC images showing cleaved caspase-3 staining in lung tissues. Scale bar, 50 μm. (*n* = 3). The results are shown as the mean ± standard error of the mean. * *p* < 0.05, ** *p* < 0.01, *** *p* < 0.001.

To elucidate andrographolide’s underlying mechanism, we performed RNA sequencing on lung tissues from IAV-infected mice with or without andrographolide treatment. Volcano plot analysis identified 4010 upregulated and 2964 downregulated genes in the andrographolide group compared with the H1N1 group (Figure 3A). Kyoto Encyclopedia of Genes and Genomes (KEGG) pathway enrichment analysis revealed a significant upregulation of the PI3K/AKT signaling pathway (Figure 3B). Gene ontology term analysis also indicated enrichment in biological processes such as the extracellular region, protein binding, and cell adhesion (Figure S2A). Western blotting validated that andrographolide treatment increased the phosphorylation levels of PI3K and AKT in mouse lung tissue (Figure 3C) and IAV-infected A549 cells (Figure 3D), confirming pathway activation.

The PI3K/AKT signaling pathway is a central regulator of cell survival, proliferation, and metabolism [51]. Its activation transmits potent anti-apoptotic and pro-survival signals. Importantly, this pathway also plays a crucial role in modulating inflammatory responses and has been specifically implicated in IAV–host interactions [52,53]. Furthermore, AKT activation is known to suppress inflammatory forms of cell death, including pyroptosis [54,55]. Therefore, the enrichment of this pathway suggested that andrographolide might exert its protective effects against IAV-induced injury by activating this key host signaling axis to enhance cellular survival and suppress inflammatory cell death. IAV infection significantly increased cleaved caspase-3 (the activated form) without altering total caspase-3, an effect that was suppressed by andrographolide (Figure 3E). Caspase-3 cleaves GSDME to generate its active N-terminal fragment (GSDME-N), which executes pyroptosis [56,57,58]. Consistent with the caspase-3 data, GSDME-N levels were significantly elevated following IAV infection, and this increase was attenuated by andrographolide treatment in vivo (Figure 3E) and in vitro (Figure 3F). The IHC assay further confirmed the reduction in cleaved caspase-3 in the lungs of andrographolide-treated mice (Figure 3G). GSDMD is a pyroptosis enforcer that can be cleaved by inflammatory caspase-1, leading to membrane pore formation [59]. The expression of cleaved caspase-1 and its substrate GSDMD, key components of the canonical pyroptosis pathway, remained unchanged upon IAV infection and andrographolide treatment both in vivo (Figure S2B) and in vitro (Figure S2C), suggesting that during IAV infection, andrographolide targets GSDME-mediated pyroptosis rather than GSDMD-mediated pyroptosis. Together, these findings indicate that andrographolide activates the PI3K/AKT signaling pathway and inhibits IAV-induced, GSDME-dependent pyroptosis.

### 3.4. The Protective Effect of Andrographolide Was Mediated Through the PI3K/AKT Signaling Pathway

**Figure 4:**
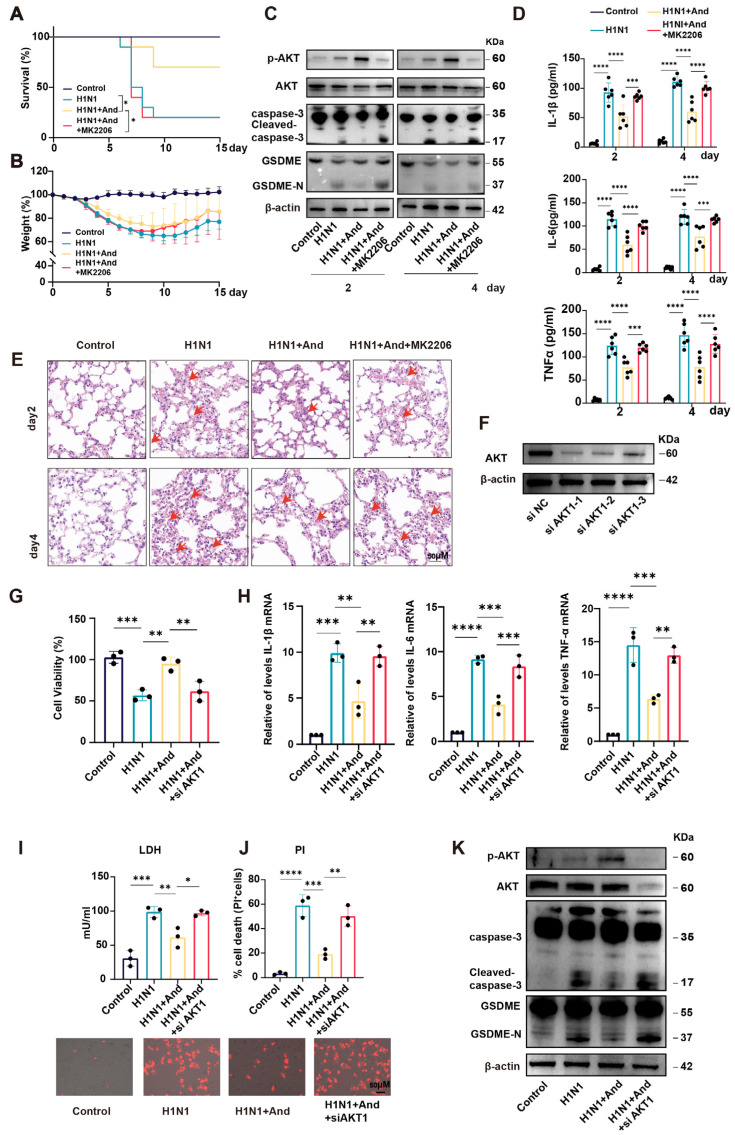
The protective effect of andrographolide was mediated through the PI3K/AKT pathway. (**A**,**B**) Mice were divided into four groups: Control (*n* = 9), H1N1-infected (H1N1, *n* = 10), H1N1-infected + Andrographolide (H1N1 + And, *n* = 10), and H1N1-infected + Andrographolide + MK-2206 (H1N1 + And + MK2206, *n* = 10). Mice in the inhibitor group were pretreated with MK-2206 (80 mg/kg, i.p.) or vehicle for 4 days before and 4 days after IAV infection. Survival rates (**A**) and body weight changes (**B**) were monitored for 15 days. (**C**) Western blotting analysis of GSDME, GSDME-N, caspase-3, and cleaved caspase-3 levels in lung tissues on days 2 and 4 post-infection. (*n* = 3). (**D**) Cytokine levels (IL-6, TNF-α, and IL-1β) in BALF were measured by ELISA on days 2 and 4. (*n* = 6). (**E**) Representative H&E-stained lung tissue sections. Arrows indicate alveolar hemorrhage and thickened alveolar walls. Scale bar, 50 µm. (*n* = 3). (**F**) Western blot analysis validating knockdown efficiency. (*n* = 3). (**G**) Cell viability of A549 cells infected with IAV and treated with siAKT1 (50 nM) and andrographolide (15 μM), assessed by CCK-8 assay. (*n* = 3). (**H**) mRNA expression levels of IL-6, TNF-α, and IL-1β in A549 cells treated with siAKT1(50 nM) and andrographolide (15 μM) post-IAV infection, measured by qRT–PCR. (*n* = 3). (**I**) LDH release was assayed in A549 cells treated with siAKT1 (50 nM) and andrographolide (15 μM) following IAV infection. (*n* = 3). (**J**) PI staining was performed in A549 cells treated with siAKT1 (50 nM) and andrographolide (15 μM) following IAV infection. Representative images of PI-stained cells were quantified. Scale bars: 50 μm. (*n* = 3). (**K**) Western blotting analysis of GSDME, GSDME-N, caspase-3, and cleaved caspase-3 levels in A549 cells infected with IAV and treated with siAKT1(50 nM) and andrographolide (15 μM). (*n* = 3). The results are shown as the mean ± standard error of the mean. * *p* < 0.05, ** *p* < 0.01, *** *p* < 0.001, **** *p* < 0.0001.

We employed the AKT inhibitor MK-2206 to directly test the functional role of the PI3K/AKT signaling pathway. The improved survival rate and attenuated weight loss conferred by andrographolide were significantly reversed by the co-administration of MK-2206 (Figure 4A,B). Accordingly, the suppression of GSDME-N and cleaved caspase-3 by andrographolide was stopped upon MK-2206 treatment (Figure 4C). The andrographolide-mediated reduction in IL-1β, IL-6, and TNF-α in BALF was also reversed by MK-2206 (Figure 4D), and lung histopathology showed more severe injury in the combination group versus the andrographolide-alone group (Figure 4E).

We also performed AKT1 knockdown using siRNA. The siRNA sequence with the highest knockdown efficiency (siRNA-1) was selected for subsequent experiments (Figure 4F). AKT1 knockdown not only reversed the andrographolide-mediated improvement in the survival of infected cells (Figure 4G) but also stopped the reduction in intracellular levels of IL-1β, IL-6, and TNF-α induced by andrographolide (Figure 4H). Furthermore, the inhibitory effect of andrographolide on virus-induced LDH release and its alleviation of cell death, as indicated by PI staining, were both abrogated upon AKT1 knockdown (Figure 4I,J). Western blot analysis further confirmed that the suppression of pyroptosis-related proteins, GSDME-N and cleaved caspase-3, by andrographolide was also dependent on AKT1 activity (Figure 4K).

In A549 cells, the andrographolide-mediated enhancement of cell viability (Figure S3A) and suppression of inflammatory cytokine mRNA expression (Figure S3B) were similarly negated by MK-2206. Western blotting confirmed that MK-2206 reversed the andrographolide-induced downregulation of cleaved caspase-3 and GSDME-N expression (Figure S3C). Notably, the combination of andrographolide and MK-2206 did not affect viral mRNA (HA, M1) or protein (HA, NP) levels in vivo or in vitro compared with andrographolide treatment alone (Figure S3D–G), confirming that the PI3K/AKT signaling pathway specifically modulates the host inflammatory response without influencing viral replication.

Overall, these results demonstrate that andrographolide alleviates IAV-induced inflammation and pyroptosis by activating the PI3K/AKT signaling pathway.

## 4. Discussion

IAV remains a significant global health threat, with severe infections often leading to pneumonia and ARDS, which are primary causes of IAV-associated mortality [1,2,3,5]. The limitations of current antiviral drugs, particularly the emergence of resistant viral strains [13], underscore the urgent need for novel therapeutic strategies. Host-directed therapies that modulate the immune response and mitigate immunopathology represent a promising approach [14,15,16,19]. In this context, natural compounds such as andrographolide, known for its broad-spectrum anti-inflammatory and antiviral properties [28,29,31,37,38,39,40,41,60,61,62,63], harbor considerable potential. However, its specific host targets and complete signaling pathways against influenza have not been fully elucidated.

Our findings demonstrate that andrographolide protects against IAV-induced cell injury and lung pathology in vitro and in vivo, without directly inhibiting viral replication, mainly by suppressing excessive host inflammation. However, there are conflicting reports on the antiviral activity of andrographolide. One research team focused on its direct antiviral effects [30], while two other independent studies (including the present work) support a host-directed mechanism [34,36], as no direct antiviral activity was observed in cellular models (Figure 2). We fully endorse the consensus that andrographolide’s primary benefit in influenza is host immunomodulation. Our work strengthens this view by providing novel mechanistic evidence through the PI3K/AKT/pyroptosis axis.

We employed RNA-seq analysis to elucidate the underlying mechanism, revealing significant PI3K/AKT signaling pathway enrichment in the lungs of andrographolide-treated mice. The PI3K/AKT pathway is a critical regulator of diverse cellular processes, including inflammation and cell survival [64]. Although this pathway has been implicated in IAV replication [52,65], our data show that andrographolide’s protection is independent of antiviral activity, suggesting a distinct, host-directed anti-inflammatory role.

We further investigated the downstream events of PI3K/AKT activation. Given this pathway’s known involvement in regulating cell death, we focused on pyroptosis, a highly inflammatory form of programmed cell death central to IAV pathology. Our results indicate that andrographolide specifically inhibits the caspase-3/GSDME axis of pyroptosis, as evidenced by reduced levels of cleaved caspase-3 and its substrate, GSDME-N. In contrast, the canonical pyroptosis pathway involving caspase-1 and GSDMD was unaffected. The critical role of the PI3K/AKT signaling pathway in this process was firmly established using the AKT inhibitor MK-2206 and siAKT1, which abolished the protective effects of andrographolide on survival, weight loss, inflammation, and pyroptosis markers both in vivo and in vitro. These findings align with previous reports that andrographolide and other compounds can exert cytoprotective effects via the PI3K/AKT signaling pathway in other disease models and extend this mechanism to the suppression of GSDME-mediated pyroptosis during viral infection [66,67,68,69].

Our study delineates a clear pathway from PI3K/AKT activation to the suppression of GSDME-dependent pyroptosis. A key question that remains is the precise molecular link between AKT activation and the inhibition of caspase-3 cleavage. We propose two non-mutually exclusive mechanisms: First, AKT could potentially phosphorylate and inhibit pro-apoptotic proteins (e.g., Bad, Bax), which might contribute to stabilizing mitochondrial integrity and thereby help prevent the apoptosome-dependent activation of caspase-3 [70,71]. Second, as a master metabolic regulator, AKT activation can promote glycolysis and metabolic homeostasis, potentially indirectly suppressing cell death by maintaining energy balance and reducing oxidative stress [72,73].

Despite the compelling evidence, there are several limitations of this study. First, the conclusions are based primarily on experiments from male BALB/c mice infected by a single mouse-adapted influenza strain (A/Puerto Rico/8/34, H1N1). Thus, whether the protective mechanism can be generalized to other influenza viral strains or to female hosts requires further investigation. Second, the pharmacokinetics and optimal formulation of andrographolide for pulmonary delivery need further optimization to enhance its bioavailability and efficacy for potential clinical application. Third, further research on the combination of andrographolide and virus-targeted drugs is needed, which might represent a promising strategy to simultaneously target the virus and mitigate immunopathology.

In conclusion, this study identifies a novel host-directed mechanism by which andrographolide alleviates IAV-induced lung immunopathology. Specifically, andrographolide protects against IAV-induced cell injury and lung pathology in vitro and in vivo without directly inhibiting viral replication, with its primary protective effect being the suppression of excessive host inflammation. Mechanistically, andrographolide activates the PI3K/AKT signaling pathway, which mediates its host-directed anti-inflammatory protection independent of antiviral activity, and specifically inhibits the caspase-3/GSDME pyroptosis axis (without affecting the caspase-1/GSDMD pathway). These findings not only deepen our understanding of andrographolide’s anti-inflammatory action but also highlight the PI3K/AKT/pyroptosis axis as a promising therapeutic target for the management of severe influenza.

Future studies will focus on elucidating the specific molecular mechanisms by which PI3K/AKT regulates the caspase-3/GSDME axis during IAV infection, verifying andrographolide’s protective effects in different IAV subtypes and clinically relevant animal models, and exploring its synergistic effects with traditional antiviral drugs as well as optimizing its administration to facilitate potential clinical translations.

## Data Availability

The original contributions presented in the study are included in the article/Supplementary Material, further inquiries can be directed to the corresponding authors.

## Supplementary Materials

The following supporting information can be downloaded at: https://www.mdpi.com/article/10.3390/biomedicines14040887/s1. Figure S1: Andrographolide reduces IAV-induced cell death. Figure S2: Andrographolide did not affect the caspase-1/GSDMD pathway. Figure S3: MK-2206 reversed the protective effects of andrographolide without affecting viral replication. Table S1: Primer sequences. Table S2: siRNA sequences.

## Abbreviations

The following abbreviations are used in this manuscript:

IAV	*Influenza A virus*
GSDME	gasdermin E
ARDS	acute respiratory distress syndrome
SARS-CoV-2	*severe acute respiratory syndrome coronavirus 2*
RIG-I	Retinoic acid-inducible gene I
NLRP3	NACHT, LRR and PYD domains-containing protein 3
STAT3	Signal Transducer and Activator of Transcription 3
Nrf2	Nuclear factor erythroid 2-related factor 2
PI3K/AKT	Phosphatidylinositol 3-kinase/AKT serine/threonine kinase
SPF	specific pathogen-free
LD_50_	median lethal dose
TCID_50_	median tissue culture infective dose
MDCK	Madin–Darby canine kidney
FBS	fetal bovine serum
PBS	phosphate-buffered saline
BALF	Bronchoalveolar lavage fluid
ELISA	enzyme-linked immunosorbent assay
OD	optical density
IHC	Immunohistochemistry
LDH	lactate dehydrogenase
PI	propidium iodide
