# Supplementary material for: Andrographolide Suppresses Influenza A Virus-Induced Pyroptosis via PI3K/AKT-Mediated Caspase-3/GSDME Inactivation
Source: Biomedicines. 2026 Apr 13;14(4):887. doi: 10.3390/biomedicines14040887 (PMC13113384; doi:10.3390/biomedicines14040887)
Supplement: Supplementary file 1 [file biomedicines-14-00887-s001.zip › biomedicines-4137219-supplementary.pdf]

Supplementary Materials for

Andrographolide Suppresses Influenza A Virus-Induced Pyroptosis via PI3K/AKT-Mediated Caspase-3/GSDME Inactivation

Wen Yang [1,†], Qi He [1,†], Zhen Sun [1], Xiaochang Zhang [1], Qingyu Li [1], Changdong Zhou [2], Yuke Cui [2], Zhenqiao Wei [1], Jingqi Shi [1], Chenhui Wang [1], Yuanyuan Jiao [1], Liang Guo [1,*], Yaling Xing [1,*] and Shengqi Wang [1,2,3,*]

1 Bioinformatics Center of AMMS, Beijing 100850, China; 15589965413@163.com (W.Y.); heqirock@163.com (Q.H.); sunzhen1710@foxmail.com (Z.S.); zhangxc@bmi.ac.cn (X.Z.); liqingyu@bmi.ac.cn (Q.L.); godbridge@outlook.com (Z.W.); jingqi_s@foxmail.com (J.S.); wangchenhuichina@tmmu.edu.cn (C.W.); jiaoyy1995@163.com (Y.J.)
2 Pingyuan Laboratory, Xinxiang 453007, China; zcd9914@163.com (C.Z.); ccccyuke@163.com (Y.C.)
3 State Key Laboratory of Kidney Diseases, Beijing 100853, China
* Correspondence: gllsunmo369@sina.com (L.G.); yalingxing@126.com (Y.X.); sqwang@bmi.ac.cn (S.W.)
† These authors contributed equally to this work.

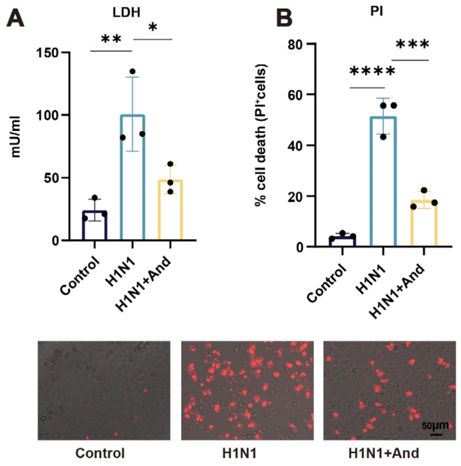

Figure S1. Andrographolide reduces IAV-induced cell death. (**A**) LDH release was assayed in A549 cells treated with andrographolide (15 μM) following IAV infection. (n = 3). (**B**) PI staining was performed in A549 cells treated with andrographolide (15 μM) following IAV infection. Representative images of PI-stained cells were quantified. Scale bars: 50 μm. (n = 3). The results are expressed as the mean ± standard error of the mean. *p < 0.05, **p < 0.01, ***p < 0.001, ****p < 0.0001.

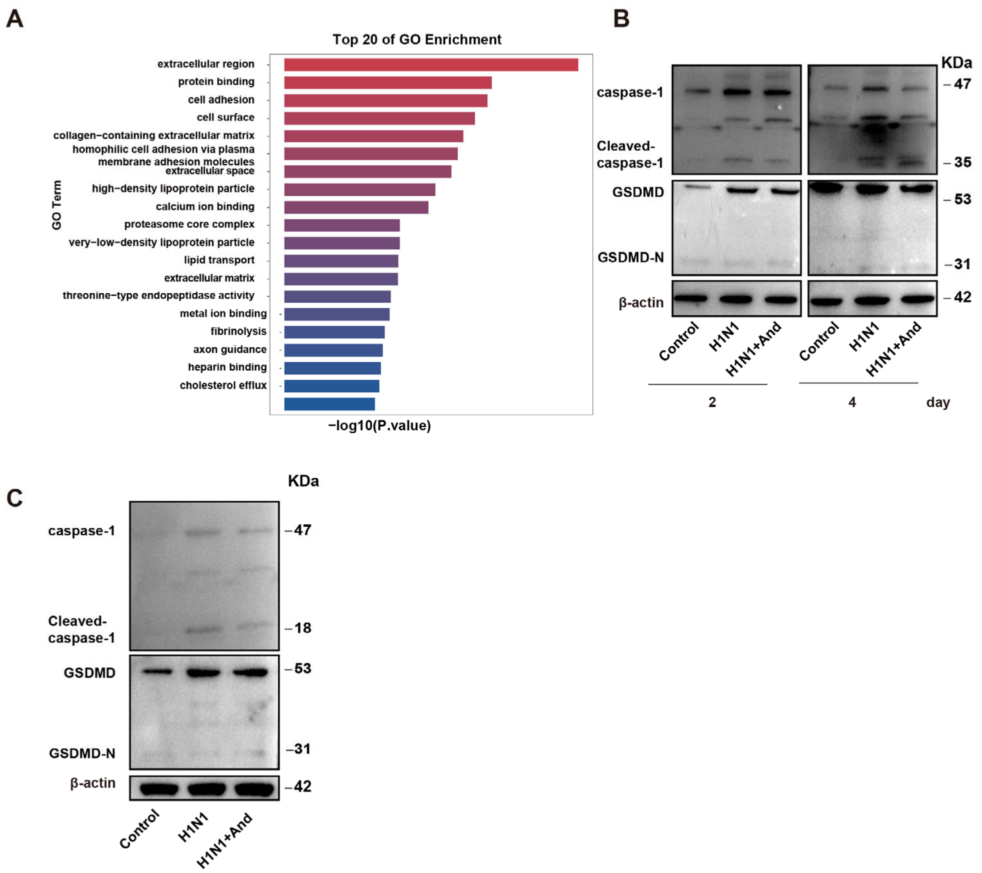

Figure S2. Andrographolide did not affect the caspase-1/GSDMD pathway. (**A**) Gene Ontology (GO) enrichment analysis of RNA-seq data from lung tissues on day 4 post-infection. (n = 4). (**B**) Western blotting analysis of GSDMD, caspase-1 protein levels in mouse lung tissues. (n = 3). (**C**) Western blotting analysis of GSDMD, caspase-1 protein levels in IAV-infected A549 cells. (n = 3).

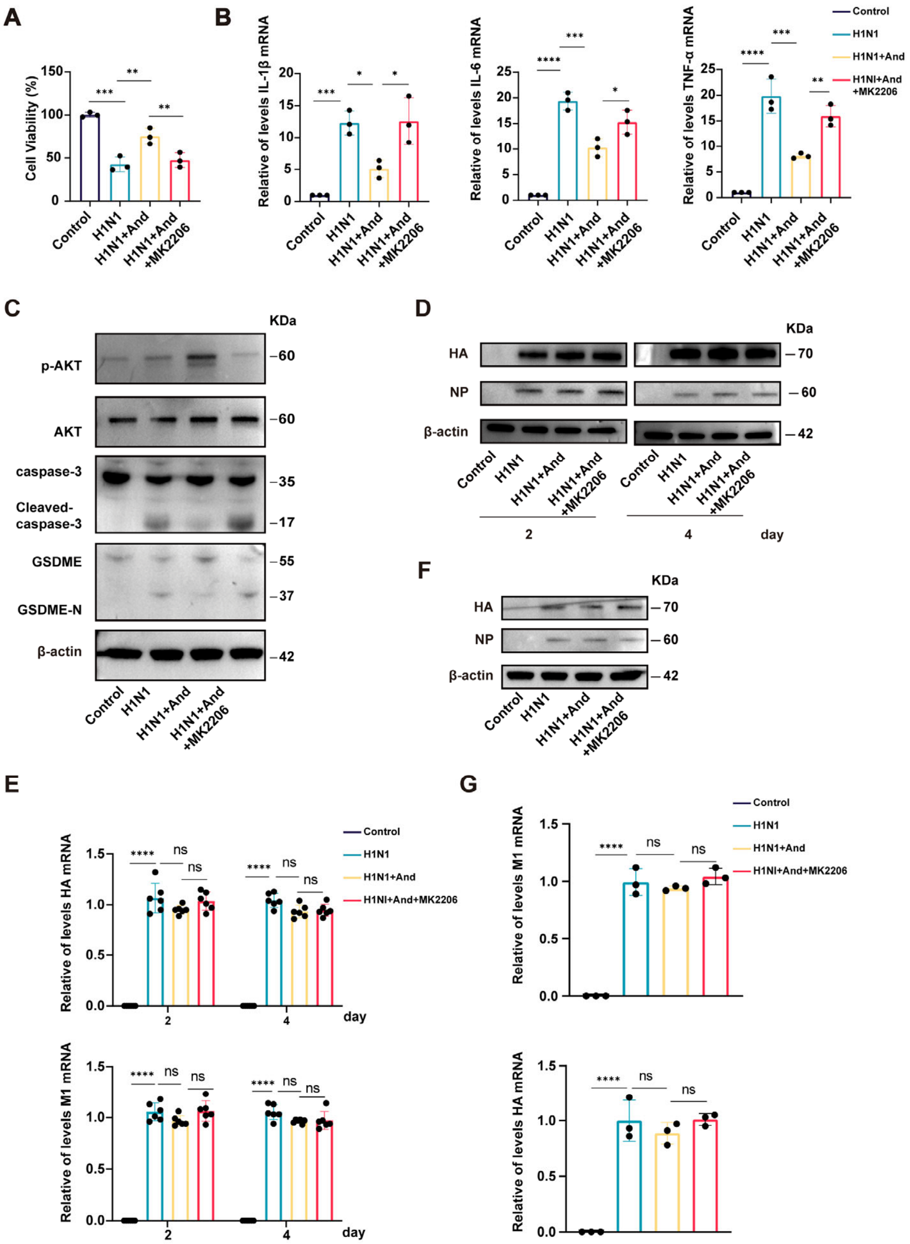

Figure S3. MK-2206 reversed the protective effects of andrographolide without affecting viral replication. (**A**) Cell viability of IAV-infected A549 cells treated with andrographolide (15 μM) and/or MK-2206, assessed by CCK-8 assay. (n = 3). (**B**) qRT-PCR analysis of IL-6, TNF-α, and IL-1β mRNA expression in A549 cells. (n = 3). (**C**) Western blotting analysis of p-AKT, AKT, GSDME, GSDME-N, caspase-3, and cleaved caspase-3 in A549 cells. (n = 3). (**D**) Western blotting analysis of viral NP/HA protein levels in mouse lung tissues from the inhibitor study. (n = 3). (**E**) qRT-PCR analysis of viral M1/HA RNA levels in mouse lung tissues from the inhibitor study. (n = 6). (**F**) Western blotting analysis of viral NP/HA protein levels in A549 cells from the inhibitor study. (n = 3). (**G**) qRT-PCR analysis of viral M1/HA RNA levels in A549 cells from the inhibitor study. (n = 3). The results are expressed as the mean ± standard error of the mean. *$p < 0.05$, **$p < 0.01$, ***$p < 0.001$, ****$p < 0.0001$. ns indicates no significance.

Table S1. Primer sequences.

Gene	*Forward*	*Reverse*
TNF-α	*5′- TCTCGAACCCCGAGTGACAA -3′*	*5′- TGAAGAGGACCTGGGAGTAG -3′*
IL-6	*5′-CGGGAACGAAAGAGAAGCTCTA -3′*	*5′- CGCTTGTGGAGAAGGAGTTCA -3′*
IL-1β	*5′- ATGGCAGAAGTACCTAAGCTC -3′*	*5′-TTAGGAAGACACAAATTGCATGGTGAACTCAGT -3′*
GAPDH	*5′- GCAAATTCCATGGCACCGT-3′*	*5′- GCCCCACTTGATTTTGGAGG-3′*
HA	*5′- AAAGAAAGCTCATGGCCCAACC-3′*	*5′- TCCTTCTCCGTCAGCCATAGCA -3′*
M1	*5′-TCATTGGGATCTTGCACTTG-3′*	*5′-ACTTTGGCACTCCTTCCGTA-3′*

Table S2. siRNA sequences.

Gene	***sense***	***antisense***
siAKT1-1	*5′- GCGUGACCAUGAACGAGUU-3′*	*5′- AACUCGUUCAUGGUCACGC -3′*
siAKT1-2	*5′- GUGGACCACUGUCAUCGAA -3′*	*5′- UUCGAUGACAGUGGUCCAC-3′*
siAKT1-3	*5′- GCCUCUGCUUUGUCAUGGA-3′*	*5′- UCCAUGACAAAGCAGAGGC-3′*
